# Interprofessional simulation of acute care for nursing and medical students: interprofessional competencies and transfer to the workplace

**DOI:** 10.1186/s12909-023-04053-2

**Published:** 2023-02-11

**Authors:** Pepijn Krielen, Malon Meeuwsen, Edward C. T. H. Tan, Jolanda H. Schieving, Annelies J. E. M. Ruijs, Nynke D. Scherpbier

**Affiliations:** 1 Department of Surgery, Radboud University Medical Center, Nijmegen, The Netherlands; 2 Department for Research in Learning and Education Radboudumc Health Academy, Radboud University Medical Center, Nijmegen, Nijmegen, The Netherlands; 3 Department of Child Neurology, Radboud University Medical Center, Nijmegen, The Netherlands; 4 Department of General Practice and Elderly Care Medicine, University Medical Center Groningen, Groningen, The Netherlands

**Keywords:** Interprofessional education, Simulation acute care

## Abstract

**Background:**

Teamwork and communication are essential tools for doctors, nurses and other team members in the management of critically ill patients. Early interprofessional education during study, using acute care simulation, may improve teamwork and communication between interprofessional team members on the long run.

**Methods:**

A comparative sequential quantitative–qualitative study was used to understand interprofessional learning outcomes in nursing and medical students after simulation of acute care. Students were assigned to a uni- or interprofessional training. Questionnaires were used to measure short and long term differences in interprofessional collaboration and communication between the intervention and control group for nursing and medical students respectively. Semi-structured focus groups were conducted to gain a better understanding of IPE in acute simulation.

**Results:**

One hundred and ninety-one students participated in this study (131 medical, 60 nursing students). No differences were found between the intervention and control group in overall ICCAS scores for both medical and nursing students (*p* = 0.181 and *p* = 0.441). There were no differences in ICS scores between the intervention and control group. Focus groups revealed growing competence in interprofessional communication and collaboration for both medical and nursing students.

**Conclusions:**

Interprofessional simulation training did show measurable growth of interprofessional competencies, but so did uniprofessional training. Both medical and nursing students reported increased awareness of perspective and expertise of own and other profession. Furthermore, they reported growing competence in interprofessional communication and collaboration in transfer to their workplace.

**Supplementary Information:**

The online version contains supplementary material available at 10.1186/s12909-023-04053-2.

## Introduction

Nurses, physician assistants and doctors often work together in teams. Team functioning demands specific skills, especially so when a patient rapidly deteriorates. In these acute situations effective interprofessional cooperation is the key factor for the success of recovery of the patient. Teams need to be aware of the (differences in) competencies, responsibilities and limits of their members. Lacking this information could negatively affect the quality of care for the patient [1–4]. The absence of effective interprofessional communication has been identified as a factor that impedes good team performance in the care of critically ill patients [5–8]. This calls for interprofessional education (IPE) in dealing with acute settings. Previous studies showed that the real-live learning environment in situations in which acute care needs to be delivered, is not optimal [9–12]. Multiple factors negatively influence optimal learning for students in these situations including: time pressure, increased workload, need for multi-tasking, unpredictability of the situation, the heterogeneity of students’ learning needs, decrease in one-on-one coaching moments and increased complexity of care [12–14].

High-fidelity simulation based learning offers time and provides a safe learning environment in which rare clinical situations can be trained [15–18]. An interprofessional approach to simulation in acute care could lead to a better mutual understanding and improved communication. This leads to the hypothesis that students may collaborate and communicate better in acute situations if trained in an IPE simulation setting [19, 20]. Simulation training showed favorable short-term effects in many studies [16–18, 21]. Long-term effects on clinical behavior of students have hardly been measured.

At the Radboud University Medical Centre and the HAN University of Applied Science, medical and nursing students, traditionally take uniprofessional classes in simulated acute care training. We developed an interprofessional simulation training for nursing and medical students, mainly based on the suggestions made by Anderson et al. on the optimal design of IPE simulation in undergraduate students [22]. This training focusses on increasing interprofessional communication, collaboration, teamwork and the insight in interprofessional team members’ qualities and pitfalls. The transfer of the acquired skills to daily clinical practice has a central role in this training. Transfer means that knowledge and skills acquired in a teaching situation can be applied in different real life situations, which is essential for young professionals [23].

The primary aim of this study was to compare and identify interprofessional learning outcomes in medical and nursing students before and after an interprofessional simulation. The secondary aim was to qualitatively and quantitatively define the long-term learning effects of an interprofessional simulation training and determine the transfer to the workplace.

## Methods

### Study design

**Fig. 1:**
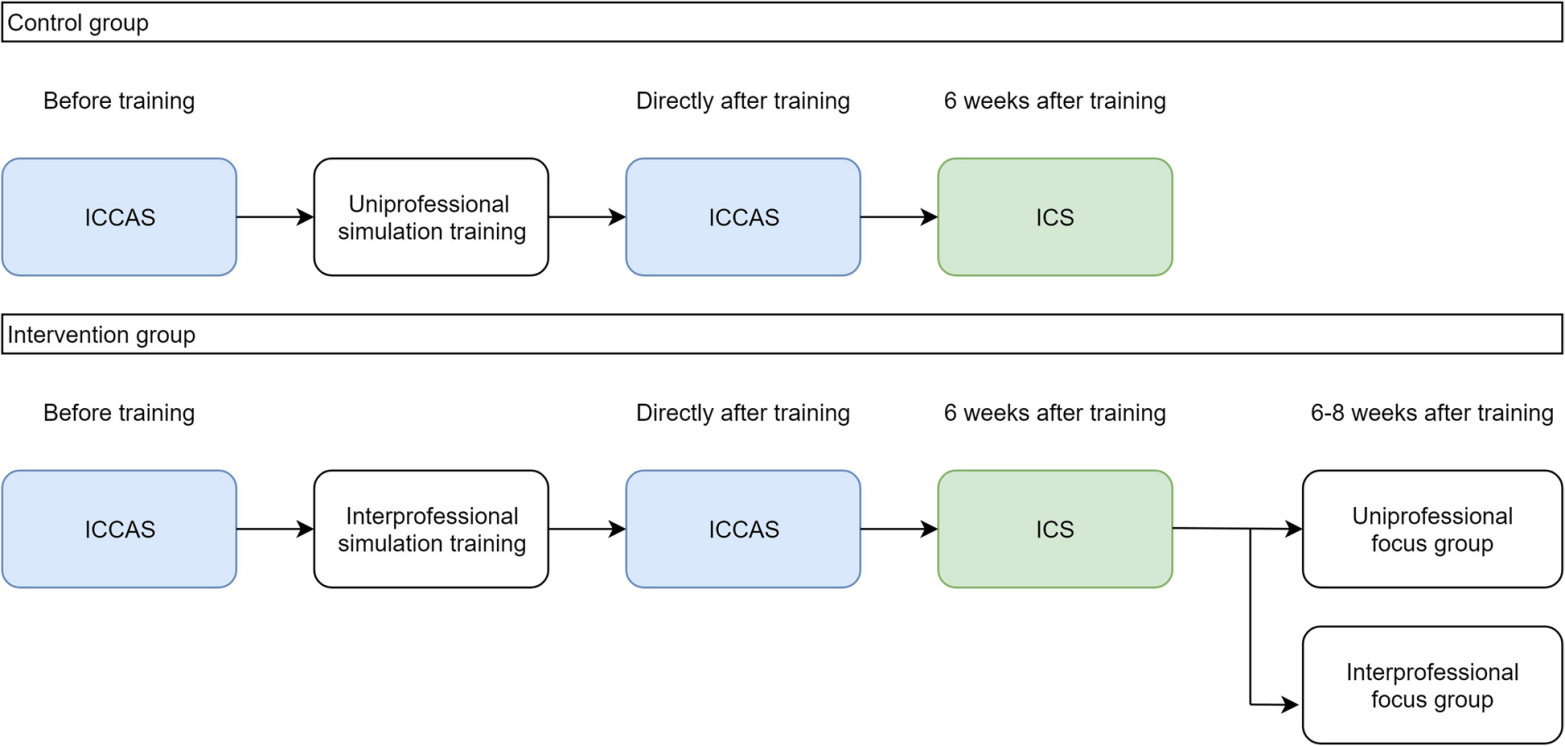
Study design

To understand the value of IPE in acute simulation education a sequential quantitative–qualitative design was used, Fig. 1. A sequential quantitative–qualitative design combines qualitative and quantitative research methods, facilitating an increase in knowledge depth and a possibility to gain a better understanding of the processes of IPE in acute medical simulation education [24]. In our study design questionnaires on interprofessional collaboration before and after the training were combined with uni- and interprofessional focus groups [25–28]. Quantitative results were compared between the intervention (interprofessional simulation) and control group (uniprofessional education).

### Participants

All last year medical and nursing students who participated in regular curricular education at the HAN University of Applied Science or at the Radboud University between August 2019 and June 2021 were eligible. In the current local education system for last year students half of all simulation training is interprofessional; due to logistic restrictions the other half is uniprofessional. For the planning of the training students were already randomly assigned to the interprofessional or uniprofessional training. In this way a control group (uniprofessional) and intervention group (interprofessional) were created pragmatically. All eligible students underwent at least one previous uniprofessional simulation training during their study.

### Setting

Simulation training consisted of a three hour training in small groups (four nursing and four medical students in case of interprofessional training, six to eight students in case of uniprofessional training). Simulation training started with an introduction in which students got to know each other and level of experience with acute situations was discussed. After the introduction students were taken to the simulation room where they got familiarized with the patient simulator. After the familiarization students were assigned to small interprofessional groups; two nurses and two doctors. During one training three different consecutive scenarios were played, all scenarios included a deteriorating patient in a hospital setting. Students who did not take an active part in the simulation could view the simulation in a different room on a monitor. Every student was an active participant in the simulation at least once. In the uniprofessional simulations for nurses the simulation ended after the nurses called for help and did a brief summary over the telephone. In the uniprofessional simulation for medical students a medical student played a non-obstructive nurse. In the interprofessional simulation nurses checked the patient and performed a structured assessment of the deteriorating patient using the ABCDE-method. When they needed help they called a doctor (medical student) and gave a brief telephonic structured summary after which the doctor entered the room and did an assessment using the ABCDE-method, treating illness as they encountered. The simulation was followed by a plenary debriefing including all students, not only the students actively participating in the simulation. The debriefing focused on interprofessional collaboration and communication, practical skills and structured analysis of an acute problem. Every IPE training was attended by two teachers, one doctor and one nurse. Uniprofessional trainings were only attended by a teacher of the students’ own faculty. All teachers were skilled in simulation training, a rotating group of ten teachers performed all trainings.

### Data collection

Quantitative data were collected using two questionnaires on IPE. The Interprofessional Collaborative Competencies Attainment Survey (ICCAS) was used directly before and after the training [25, 26, 28]. ICCAS is a self-reported competency survey aimed toward interprofessional collaboration. It comprises 21 questions using a 7-point scale (1 = strongly disagree, 7 = strongly agree). The subdomains for the ICCAS (communication, collaboration, roles and responsibilities, collaborative patient/family-centered approach, conflict management/resolution, team functioning) align with the learning outcomes of the interprofessional simulation as set by the authors [25, 26]. A validated Dutch version of the ICCAS was available and used for this study. The ICCAS was designed to assess changes in interprofessional collaboration competencies directly before and after IPE training interventions, and was therefore not suited to measure the long-term effect of the training.

Six weeks after the training an electronic version of the Interprofessional Collaboration Scale (ICS) was filled in by all participants [27]. The ICS was used as a tool to measure the long-term interprofessional collaboration effect of the simulation training. The ICS is a self-reported survey on the perception of interprofessional collaboration of nurses and doctors. It holds 13 questions using a 4-point scale (1 = strongly disagree, 4 = strongly agree). Three subscales are measured in the ICS; communication, accommodation and isolation (“they do not ask for our opinions”, “they think their work is more important than the work of us”). As no validated Dutch version of the ICS was available, with help of a native English speaker we used back- and forth-translation to create a Dutch version of the ICS.

Qualitative data were collected six to eight weeks after the interprofessional training in semi-structured focus group discussions exploring interprofessional learning outcomes (Additional file 1: Appendix). To encourage interaction between students interprofessional focus groups were held in which students were invited to discuss their learning outcomes with the other profession. To take into account the possibility that the interprofessional character of the focus groups might overwhelm some students resulting in a decrease in interaction, we also held uniprofessional focus groups. Students were randomly assigned to a uni- or interprofessional focus group. All focus groups were audiotaped. Audiotaped data were transcribed and anonymized.

### Data analysis

Differences in ICCAS scores before and after the training were displayed as delta and compared between intervention and control group for medical and nursing students. ICS scores were compared between the intervention and control group for medical and nursing students.

Quantitative data were analyzed using statistical program IBM Statistical package for Social Sciences (SPSS, Chicago, II, 2018) version 25.0 with a significance threshold of *p* < 0.05. Comparison between groups was made using Chi-square, Fisher’s exact test, independent t-test or Mann–Whitney U test where appropriate. Normally distributed continuous variables are presented as means with standard deviation, in case of non-normal distribution median with interquartile range (25–75) is presented. Dichotomous or categorical data are presented as absolute numbers and percentages.

Qualitative analysis was conducted based on the six-step approach of Braun and Clark [29]. Thematic analysis was based on an essentialist approach, theorizing experiences, meanings and the reality of participants in a straightforward way. Each focus group was at least coded by two researchers out of four (AR, MM, NS, PK). All focus groups were read and reread by the researchers before we started the coding, in order to get familiarized with the data. We started with initial coding using an open coding system, adapting and refining the framework with each new focus group analysis. After analyzing three focus groups a definitive version of the coding framework was constructed. The remaining focus groups were coded using the definitive version of the framework. Afterwards the first three focus groups were coded again using the definitive framework. The two initial researchers discussed their coding scheme and agreed on final coding. In case of disagreement a third researcher was asked for his opinion. All focus groups were coded in a phased fashion, interim analysis was conducted to check for saturation. After coding NS, AR and PK wrote all codes on a separate piece of paper and organized them through discussion into coherent patterns. The final stage consistent of reviewing and refining the patterns and constructing and naming definitive themes.

### Ethical approval

Ethical approval for this study was granted by the Ethical Review Board of the Dutch Authority on Medical Education. All participants were informed on this study using a plain language information sheet outlining the study and signed an informed consent before the start of the study.

## Results

A total of 196 medical students and 104 nursing students were eligible for inclusion. Demographics are presented in Table 1; there were no baseline differences between the control and intervention group for both medical and nursing students.

**Table 1:** Demographics of study population, mean (SD) or N (%)

	**Nursing students**			**Medical students**		
	Control (*N* = 41)	Intervention (*N* = 33)	Sig.	Control (*N* = 67)	Intervention (*N* = 64)	Sig.
Age (year)	21.5 (2.8)	21.4 (1.2)	*P* = 0.941	24.4 (1.5)	25.0 (2.5)	*P* = 0.083
Gender (female)	35 (85.4%)	29 (87.9%)	*P* = 0.753	48 (71.6%)	37 (57.8%)	*P* = 0.097

### ICCAS

Two hundred and five students completed the ICCAS questionnaires before and after the training (medical = 134 (68.4%), nursing = 74 (71.2%). Both medical and nursing students scored better on all subdomains of the ICCAS after the training compared with baseline (communication, collaboration, roles and responsibilities, collaborative approach, conflict management and team functioning). Comparison of the control and intervention groups for medical and nursing students showed no differences in total ICCAS scores. Analysis of subdomains of the ICCAS showed a significant increase in learning outcomes on ‘Roles and Responsibilities’ for nurses in the intervention group compared with the control group (ICCAS Roles and Responsibilities score 1.55 (SD 2.27) vs 0.56 (1.79), *p* = 0.040). None of the other subdomains of the ICCAS showed significant differences between the intervention and control groups (Table 2).

**Table 2:** ICCAS scores before and after training, mean (SD)

		**Nursing**			**Medical**		
		Control (*N* = 41)	Intervention (*N* = 33)	Sig.	Control (*N* = 67)	Intervention (*N* = 67)	Sig.
ICCAS total	Before	65.63 (7.52)	69.42 (8.12)	***P* = 0.041**	69.18 (5.06)	65.81 (6.47)	***P* = 0.021**
	After	69.68 (7.56)	75.39 (8.87)	***P* = 0.004**	75.37 (5.81)	71.95 (8.28)	***P* = 0.007**
	Diff	4.05 (5.48)	5.97 (6.76)	*P* = 0.181	7.19 (5.65)	6.14 (9.39)	*P* = 0.441
ICCAS Communication	Before	15.59 (1.72)	16.18 (2.27)	*P* = 0.202	16.55 (1.76)	15.97 (1.93)	*P* = 0.073
	After	16.93 (2.14)	18.30 (2.21)	***P* = 0.008**	18.63 (2.02)	18.11 (2.22)	*P* = 0.165
	Diff	1.34 (1.74)	2.12 (1.93)	*P* = 0.072	2.07 (1.92)	2.14 (2.64)	*P* = 0.871
ICCAS Collaboration	Before	10.51 (1.61)	11.36 (1.41)	***P* = 0.020**	10.57 (1.21)	10.25 (1.49)	*P* = 0.183
	After	11.07 (1.49)	11.82 (1.53)	***P* = 0.038**	11.60 (1.02)	11.16 (1.70)	*P* = 0.083
	Diff	0.56 (1.23)	0.45 (1.39)	*P* = 0.728	1.03 (1.33)	0.91 (2.04)	*P* = 0.684
ICCAS Roles and responsibilities	Before	13.32 (2.03)	13.76 (2.08)	*P* = 0.362	13.57 (1.68)	13.13 (1.72)	*P* = 0.140
	After	13.88 (1.66)	15.30 (2.13)	***P* = 0.002**	15.33 (1.55)	14.41 (1.85)	***P* = 0.002**
	Diff	0.56 (1.79)	1.55 (2.27)	***P* = 0.040**	1.76 (2.32)	1.28 (2.58)	*P* = 0.264
ICCAS Collaborative approach	Before	10.15 (1.71)	11.24 (1.84)	***P* = 0.010**	10.70 (1.38)	10.05 (1.48)	***P* = 0.010**
	After	10.59 (1.52)	11.58 (1.85)	***P* = 0.014**	11.07 (1.26)	10.61 (1.58)	*P* = 0.066
	Diff	0.44 (1.43)	0.33 (1.73)	*P* = 0. 774	0.37 (1.57)	0.56 (1.85)	*P* = 0.528
ICCAS Conflict management and team functioning	Before	16.08 (2.50)	16.88 (2.60)	*P* = 0.180	16.79 (1.83)	16.43 (2.22)	*P* = 0.301
	After	17.22 (2.19)	18.39 (2.70)	***P* = 0.042**	18.75 (1.74)	17.67 (2.36)	***P* = 0.004**
	Diff	1.15 (2.13)	1.52 (2.24)	*P* = 0.471	1.96 (2.29)	1.25 (3.03)	*P* = 0.134
Improvement of interprofessional collaboration after the training	After	3.51 (0.68)	3.76 (0.61)	*P* = 0.110	3.94 (0.34)	3.97 (0.62)	*P* = 0.746

*ICCAS* Interprofessional Collaborative Competencies Attainment Survey, *SD* Standard deviation, *Diff* Difference, a positive value indicates an increase in ICCAS score after the training

### ICS

Twelve nursing students (11.5%) completed the ICS questionnaire, six from the control and six from the intervention group. Sixty medical students (30.6%) completed the ICS questionnaire, 42 in the control group and 28 in the intervention group. No difference in total ICS scores or in any of the three subdomains was found between the control and intervention group for both medical and nursing students (Table 3).

**Table 3:** ICS scores per profession, mean (SD)

	**Nursing**			**Medical**		
	Control (*N* = 6)	Intervention (*N* = 6)	Sig.	Control (*N* = 42)	Intervention (*N* = 28)	Sig.
ICS total	35.33 (3.61)	34.67 (1.63)	*P* = 0.689	33.93 (1.67)	34.50 (1.77)	*P* = 0.176
ICS communication	13.17 (1.17)	12.67 (0.82)	*P* = 0.411	13.48 (0.94)	13.43 (1.03)	*P* = 0.843
ICS accommodation	13.83 (1.47)	15.00 (1.55)	*P* = 0.211	14.76 (1.54)	15.14 (1.38)	*P* = 0.295
ICS isolation	8.33 (2.50)	7.00 (0.89)	*P* = 0.263	5.69 (0.87)	5.93 (0.77)	*P* = 0.244

*ICS* Interprofessional Collaboration Scale, *SD* Standard deviation

### Qualitative results

Qualitative analysis of the focus groups identified four key themes: increasing awareness of perspective and expertise of own and other profession, growing competence in interprofessional communication and collaboration, developing professional identity and challenging implicit assumptions and reducing hierarchy.

#### Increasing awareness of perspective and expertise of own and other profession

##### Perspective

For many students the simulation was the first time they actively engaged with the other profession. Previously they have met on the ward but rarely had time or opportunity for a more elaborate communication. Students found it enlightening to learn more about ‘where the medical students are in their studies and how they think’ (Nursing student) and ‘what goes on in the head of the nurses, what do they see, what are their considerations’ (Medical student).

Medical students stated that they became more aware of the value of the nurses’ perspective.

Medical student: *‘They spend so much more time with the patient than us doctors and notice the smallest changes. So when they call and say ‘could you come and take a look because I don’t trust it’, you’d better take that very seriously.’*

Nursing students realized how challenging it is for a doctor/medical student to enter an acute situation with an unknown patient and how important the nurses’ own perspective and expertise is in informing them.

Nursing student: *‘I saw how hard it was for the medical student to know what to do based on our briefing over the phone. So even when he is in the room our role still is very important; to state again what we have seen and what we see now. We are the link between the patient and the doctor.’*

##### Expertise

The simulation gave medical students the opportunity to find out more about the expertise of the nursing students. They found that nurses had much more practical knowledge, about IVs and oxygen, than they previously realized.

Nursing students appreciated to get more insight in the role and expertise of the doctor and to experience how the professions complement each other. Nursing student: *‘One is not better than the other, but where my knowledge stops, theirs comes in. ‘*

#### Growing competence in interprofessional communication and collaboration

By practicing an acute situation together and having a debriefing shortly after, students became aware of the importance of explicit and responsive communication.

Medical student: *‘It is not that the nurse follows orders and you do the thinking. We were encouraged to think out loud so the nurse could follow our thoughts and we knew what the nurse was thinking. That makes collaboration easier.’*

Nursing student: ‘*The training demonstrated the importance of speaking the same language. We found out that they didn’t know some of the terms we used and *vice versa*. So in our communication to the doctors we need to be clear, avoid abbreviations, and use closed loop communication.’*

Students also learned how important it is to verify that you were understood.

Nursing student: *‘In our simulation the patient wasn’t doing so well. The nurses had suggested to call for assistance and we thought the doctor understood. In the debriefing we learned that we should be more insistent, explicitly ask: did you understand me? But as a nurse I tend to be a bit insecure and I think: she is the doctor, she knows best.’*

##### Transfer

The focus interviews were conducted six to eight weeks after the training. Students reported transfer of IPE competencies in their work on the wards.

Medical student: *‘It is now easier for me to delegate, to give clear and timely orders and then it will be all right. And also, I actively ask for the nurses opinion.’*

Nursing student: *‘In the training the medical students told me to turn my briefing around. First start with: I have a patient with this or that, and I would like you to come, and then tell the rest. The doctor then listens differently because the urgency is clear. So now I use that when I call.’*

#### Developing professional identity

Another effect of the simulation was that nursing students developed a stronger professional identity in the sense that they became more aware (and felt proud) of their own role and expertise.

Nursing student: ‘*Beforehand I was a bit apprehensive of the simulation. What if I don’t know what I am doing? But then it turned out we nurses actually know quite a lot and are not afraid to act and speak out. So I was kind of proud to show that to the medical students.’*

Nursing student: *‘I noticed that in the acute situation, we are the ones that communicate with the patient. The doctors were probably very busy in their head with the diagnosis so we said things to the patient like: okay sir, the doctor will now feel your abdomen. We stood closer to the patient and noticed first when he deteriorated.‘*

For some nurses the training motivated them to take an initiative in their internship to improve interprofessional collaboration.

Nursing student: *‘Since the training I have re-introduced the ABCDE method just to be certain that I didn’t miss anything. The doctors sometimes give me a funny look but I think that clear communication is important in any situation. And I am convinced that a more structured round saves time in the end. So I just stick to it.’*

Nursing student: *‘The other day we had an emergency with a breathing cannula and the emergency trolley wasn’t adequately equipped for it. So now I am going to prepare a clinical lesson about it. The training taught me how important it is that you keep your knowledge up to date and to use each other’s knowledge.’*

#### Challenging implicit assumptions and reducing hierarchy

Despite improvements in the nurse–doctor relationship over the last few decades, nursing often continues to be socially positioned and understood as inferior to medicine. By working together in the acute simulation the medical students found that their implicit assumptions about the role and expertise of the nurses were challenged.

Medical student*: ‘Of course in a way I already knew that before they call you, nurses have already done a lot, and thought about what the problem could be, but the training made it more concrete.’*

The nursing students valued the simulation for the opportunity it offered to get to know medical students more personally. This had a positive effect on the perceived hierarchy between the professions.

Nursing student:* ‘Traditionally, the hospital is rather hierarchical. But the training makes that disappear because you get to know each other more personally. When you share your experiences and what you are insecure about, it lowers the threshold’.*

Nursing student: *‘In the hospital you sometimes get the impression that doctors think themselves high and mighty but that is not the case with this generation. They really value to hear everyone’s opinion.’*

#### Organizational aspects

Overall students really appreciated the training. They found it educational, useful and fun. The timing of the training (before their last internship) was considered adequate because now they had much more responsibilities and could actually get into the situation the training depicts. They recommend to make the training available for all students in their last year.

## Discussion

The interprofessional simulation training was associated with an increase in ICCAS scores on all subdomains for both nursing and medical students. However, no differences in ICCAS scores between the control and intervention group were found. No differences between the intervention and control group were observed in ICS scores six weeks after the training.

The lack of differences in ICCAS scores between the intervention and control group might be partly explained by the fact that the students in the uniprofessional training also increased their awareness of the perspective and capabilities of the other profession through roleplay. For example, during uniprofessional training part of the medical students take on the role of the nurse, providing insight in the role of the nurse in an interprofessional team. Furthermore, both the intervention and the control group students were coached by a teacher to give feedback on their own strengths and shortcomings. Before the training the students might be unconsciously incompetent while after the training they became consciously incompetent or even consciously competent.

The sequential quantitative–qualitative design of this study provided us the opportunity to further explore the learning outcome in focus groups. Focus groups with students of the intervention group revealed an increasing awareness for both medical and nursing students of the perspective and expertise of their own and the other profession. Both also reported growing competence in interprofessional communication and collaboration. Nursing students in particular told they gained growing insight in their role in acute situations; they became more proud of their own profession. Medical students realized the added value of a nurse in acute situations, and both groups said they improved their communicative skills during the interprofessional training. After the training both medical and nursing students indicated that the growing competence in interprofessional communication also reduced hierarchy, which in turn ensures better interprofessional collaboration.

### Strengths and limitations

One of the major strengths of this study is the comparison between the intervention group and a control group. Most studies on IPE simulation only report the effect for the intervention group. Furthermore, effects of the interprofessional simulation training were not only measured directly after the training but also after 6 weeks to gain insight in the long-term interprofessional collaboration effects.

Another strength of this study is the use of a sequential quantitative–qualitative design measuring the effect of the interprofessional simulation training. It is not clear whether the self-assessed improvements in interprofessional collaboration and communication alone are associated with growing interprofessional collaboration in real-life [22, 28]. Medical professionals often tend to overrate or underestimate their skills during self-assessed surveys, which is called the Dunning-Kruger effect [30, 31]. The Dunning-Kruger effect states that low performers do not know that they are low performers and overestimate their abilities [31]. The undergraduate students in this study rates themselves good to very good on the ICCAS scale before the training, while having little to no experience with interprofessional collaboration, indicating overconfidence. The use of focus groups to gain a better understanding of the complex learning situation in IPE simulation results in a deeper understanding than using a solely quantitative analysis.. Studying complex learning situations might therefore be better studied using qualitative methods, i.e. focus groups or direct observations [22, 28].

A few limitations of our study need to be discussed. We used the ICS questionnaire to gain insight in the potential real-live improvements in interprofessional collaboration and communication of the participants. The response rate for this questionnaires was unfortunately low in this study; as a result no definitive conclusions can be made on the long-term real-life learning effect of our training. Further research should focus on the validity and transfer of learned skills to the workplace, ideally in a study based on expert based observations in real-life situations. We did not perform focus groups with students from the uniprofessional training, as we were looking for experiences from the interprofessional group.

Simulation training provides a safe environment in which rare clinical situations can be trained [15–18]. Teams are able to practice essential interprofessional communication and collaborative skills, and explore their professional role and responsibility [32, 33]. Short-term learning effects of IPE acute care simulation training are favorable in many studies, including increased confidence after interprofessional training [16–18, 21, 34, 35]. However, the long-term retention of learned abilities and their effect on the behaviors of students in the clinical setting are often not measured. The duration and frequency of these kind of trainings is therefore debatable. Ideally IPE training would be done often, however costs of IPE simulation training are relatively high. In a study by Data et al. teamwork skills improved after two simulation scenario’s during ten months, showing an increase in teamwork compared with baseline [36]. Another study on self-assessed confidence in the improvement of technical skills after an interprofessional training reported declining confidence three to five year after the training [34]. Optimal interprofessional training may therefore be best organized in yearly or half yearly episodes, resulting in increased knowledge and skills and retaining learned abilities.

Results for our study were comparable to other studies in which interprofessional communication training improved self-confidence and perception for both nursing and medical students [21, 36]. Results of previous studies were however not compared with a control group; our study design facilitated a comparison between medical and nursing student who followed a uni- or interprofessional simulation training. Unfortunately quantitative comparison of results between the control and intervention group showed no differences in learned interprofessional capabilities. This raises the question whether the organization of interprofessional simulation is worth the effort compared to uniprofessional simulation. The results of the qualitative part of this study give show that students report positive learning outcomes and transfer to the workplace after interprofessional simulation training.

## Conclusion

IPE simulation training provides a useful tool for both medical and nursing students in learning about each other’s strengths and limitations in acute care and increase their interprofessional communication and collaboration skills. Although we could not measure a difference in growth of interprofessional competencies compared to uniprofessional training, both medical and nursing students reported a growth in the awareness of perspective and expertise of their own and the other profession. Nursing students became more proud of their own profession as they gained insight in their role in acute situations. Medical students realized the added value of a nurse in acute situations.

## Supplementary Information

**Additional file 1:**

## Data Availability

The datasets generated and/or analysed during the current study are not publicly available due privacy restrictions but are available from the corresponding author on reasonable request.
