# Supplementary material for: Interprofessional simulation of acute care for nursing and medical students: interprofessional competencies and transfer to the workplace
Source: BMC Med Educ. 2023 Feb 11;23:105. doi: 10.1186/s12909-023-04053-2 (PMC9921059; doi:10.1186/s12909-023-04053-2)
Supplement: Supplementary file 1 — Additional file 1: [file 12909_2023_4053_MOESM1_ESM.docx]

**Appendix**

# Example of a scenario used in the simulation training

**Setting**Pulmonary ward

**Casus**Eighty year old man, admitted 5 days ago with a pneumonia, was treated with iv antibiotics (ceftriaxone 2000mg 1dd). Due to a decreased urinary output patient had several fluid challenges, to closely monitor his urinary output patient had a catheter. Patient recovered well and can be discharged today. Yesterday his iv and catheter were removed.

Medical history: COPD gold II, diabetes mellitus type 2

*Medication: insuline, short-acting bronchodilator inhalers*

**Scenario**

You (the nursing student) took care of the patient the last few days and saw him recover. The nurse from the nightshift told you that he was a little bit disoriented during the night and saw ants walking on the wall during the night. You start your shift and will go check the patient.

When the nurses enter the patient room, patient is complaining of pain in his lower abdomen and is talking and does not now where he is.

The nurses do a short anamnesis and start with the ABCDE. Patient did not go to the toilet since the catheter was removed. Patient deteriorates. Upon finding the patient in shock, the nurses call the doctor (medical student) and give a brief structured summary based on the RSVP/SBAR.

The doctor then enters the scenario and together with the nurse they repeat the ABCDE, treating all deviations found. Patient has urinary retention and develops a severe sepsis which needs treatment with iv fluids and antibiotics. If the doctor needs help he can call his/her supervisor with the telephone present in the room.

**Goals**

*Technical skills*

- Using the ABCDE structure to asses a critically ill patient (nursing and medical student)
- Recognizing sepsis (nursing and medical student)
- Starting oxygen therapy and fluid resuscitation (nursing and medical student)
- Starting antibiotic therapy (medical student)

*Non-technical skills*

- Get help (nursing and medical student)
- Using the SBAR/RSVP structure to give a brief summary on the telephone (nursing and medical student)
- Closed loop communication (nursing and medical student)

| **Stage** | **Simulator** | **Patient response** | **Goals** |
| --- | --- | --- | --- |
| **Stage 1**  **Start scenario** | RR 20/min  SpO2 95%  HR 105/min  RR 102/60 mmHg  Temp 38.8 ^o^C | A: talks, trachea in midline  B: normal auscultation  C: CR 3 sec, acra cool, abdomen tense, pain in the lower abdomen  D: E4M5V6, PEARL, glucose 7.2  E: pale | Taking vital signs  Systematic assessment of the critically ill patient using ABCDE |
| **Stage 2**  **Increased sepsis** | RR 🡩 25 /min  SpO2 🡫 88% (with NRM 93%)  HR 120/min  RR 80/ 50 mmHg  Temp 39.5 ^o^C | A/B: no changes  C: CR 4 sec, acra cool, increased abdominal pain  D: responds to pain  E: temp 39.5 | Starting oxygen therapy an iv fluids  Starting iv antibiotics  Thinking about lab works or the need of radiological imaging studies  When a catheter is placed 600cc cloudy urine is in the bag |
| **Stage 3**  **Recovery** (when iv antibiotics and fluid resuscitation is started) | RR 20/min  SpO2 98%  HR 80/min  RR 110/80 mmHg  Temp 38.5 ^o^C | Patient is agitated but luicid | Reassessment of ABCDE |

# Interview Guide IPE Focus Groups

**Instruction focus group leader**

- The interview guide below is a guideline for the interview with the students that participated in the interprofessional simulation training.
- Please ask open questions.
- Invite students to elaborate on the experiences they describe.
- Check whether other students recognize the experiences described.

**Introduction (read this verbatim)**

The next 45 minutes we are going to talk about your experiences with the interprofessional simulation training and whether you were able to incorporate the things you learned in your internships.

My role is to lead the discussion. I am interested in your experiences and would like to encourage you to talk to each other, ask each other questions and discuss what has been said. There are no wrong answers.

Please take care to talk in turn and not all at once.

- This conversation is recorded. After transcriptions, the audio files will be destroyed.
- All information is anonymized and will not be traceable to a person.
- This focus group is confidential.
- Nothing that will be said here will influence your grades.

**Introductions**

Make a round of introductions, with names, role in the internship, year of study.

**Start of the focus group**

We will now start with the focus group. To be able to answer our research question, it is important to keep this question in your mind: How do nursing and medical students view the added value of the interprofessional simulation training for their learning and working on the wards?

**Start recording.**

| **Question** |  | **Purpose** |
| --- | --- | --- |
| **1** | What did you think of the training? | Discuss first impressions, get students going. |
| **2** | What have you learned through the training?  *Examples of additional questions:*  *Does it have an added value? What made it educational for you? How did you experience the training? What did you do with your experiences at the workplace? How is that reflected in your actions?* | Orientation on relevant experiences |
|  |  | Further clarify experiences, ask additional questions when relevant |
| **3** | What have you learned about your own profession during and after the training? | Clarify learning outcomes |
| **4** | What have you learned about the other profession during and after the training? | Clarify learning outcomes |
| **5** | What have you learned about the way the other profession perceives you? | Clarify learning outcomes |
| **6** | Have you purposely used elements from the training in your internships? Or were there elements from the training that you have deliberately NOT used?  *Examples of additional questions:*  *Can you illustrate this with an example? How would this be visible in your actions? Why did you choose to apply these specific elements in your internship?* | Explore transfer to internship |
| **7** | Are there any other aspects that you intend to change in your future work? | Identify future intentions |
| **8** | Are there any other things you would like to share on this subject? Something you perhaps expected me to ask? Something that has not been mentioned yet? |  |
|  | Thank participants. |  |
